# The Role of the Clinically Obtained Acoustic Reflex as a Research Tool for Subclinical Hearing Pathologies

**DOI:** 10.1177/2331216520972860

**Published:** 2020-12-24

**Authors:** Andrew Causon, Kevin J. Munro, Christopher J. Plack, Garreth Prendergast

**Affiliations:** 1 Manchester Centre for Audiology and Deafness, School of Health Sciences, University of Manchester, UK; 2 Manchester Academic Health Science Centre, Manchester University NHS Foundation Trust, Manchester, England; 3 Department of Psychology, Lancaster University, Lancaster, England

**Keywords:** cochlear synaptopathy, hidden hearing loss, acoustic reflex, middle-ear muscle reflex

## Abstract

The acoustic reflex (AR) shows promise as an objective test for the presence of cochlear synaptopathy in rodents. The AR has also been shown to be reduced in humans with tinnitus compared to those without. The aim of the present study was twofold: (a) to determine if AR strength (quantified as both threshold and growth) varied with lifetime noise exposure, and thus provided an estimate of the degree of synaptopathy and (b) to identify which factors should be considered when using the AR as a quantitative measure rather than just present/absent responses. AR thresholds and growth functions were measured using ipsilateral and contralateral, broadband and tonal elicitors in adults with normal hearing and varying levels of lifetime noise exposure. Only the clinical standard 226 Hz probe tone was used. AR threshold and growth were not related to lifetime noise exposure, suggesting that routine clinical AR measures are not a sensitive measure when investigating the effects of noise exposure in audiometrically normal listeners. Our secondary, exploratory analyses revealed that AR threshold and growth were significantly related to middle-ear compliance. Listeners with higher middle-ear compliance (though still in the clinically normal range) showed lower AR thresholds and steeper AR growth functions. Furthermore, there was a difference in middle-ear compliance between the sexes, with males showing higher middle-ear compliance values than females. Therefore, it may be necessary to factor middle-ear compliance values into any analysis that uses the AR as an estimate of auditory function.

Cochlear synaptopathy is part of presbycusis, the natural aging process of hearing (Wu et al., 2018), as well as a consequence of traumatic events such as exposure to intense sounds (Kujawa & Liberman, 2009). Although the auditory brainstem response (ABR) wave I amplitude is a good biomarker for the presence of synaptic loss in numerous rodent models (see Hickox et al., 2017 for a review), the evidence for noise-induced synaptopathy in humans is less clear (Le Prell, 2019). Some studies report that wave I amplitude (Bramhall et al., 2017), wave I/V ratio (Grose et al., 2017), and the action-potential/summating potential ratio (Liberman et al., 2016) are decreased for noise-exposed listeners despite normal audiometric hearing in the clinical frequency range. However, a large number of studies also show no relation between suprathreshold ABR amplitudes and noise exposure (Fullbright et al., 2017; Grinn et al., 2017; Guest et al., 2017; Prendergast et al., 2017; Smith et al., 2019; Spankovich et al., 2017).

Valero et al. (2016, 2018) found that the acoustic reflex (AR) threshold is a sensitive measure of cochlear synaptopathy in noise-exposed mice, even more so when the elicitor is constrained to the same spectral region as the initial noise insult. Wojtczak et al. (2017) performed a study in human listeners with normal audiometric thresholds and either with or without tinnitus. The AR was measured with a broadband noise (BBN) eliciting the response at six presentation levels (63–88 dB sound pressure level [SPL]) and measured with a broadband click probe in the contralateral ear. The AR in the tinnitus group was dramatically weaker (almost absent) than the control group and showed significantly weaker growth over the tested range. Wojtczak et al. (2017) suggested that the weaker response in listeners with noise-induced tinnitus could be due to a loss of cochlear synapses. The rationale is based on rodent work which found the loss of synapses in noise-exposed rodents to be largely in a subset of auditory nerve fibers with high thresholds and low rates of spontaneous activity (Furman et al., 2013; Kujawa & Liberman, 2015). A selective loss of only high-threshold fibers would not affect audiometric thresholds but could affect hearing in noisier environments. Therefore, Valero et al. (2016) propose that the AR is an excellent candidate for studying synaptopathy because the AR recruits neurons with high thresholds. This has led to renewed interest in the AR as an investigative, quantitative tool, in concordance with much earlier studies showing its suitability for detecting changes in the human auditory system in the absence of audiometric threshold elevation (Celik et al., 1998; Gerhardt & Hepler, 1983).

Guest et al. (2019) performed a study in which AR thresholds were measured ipsilaterally using tonal elicitors. The research investigated the relation of the AR threshold to tinnitus, speech-in-noise ability, and self-reported noise exposure. None of the dependent variables measured were related to AR threshold, with the lack of difference in AR threshold as a function of tinnitus contrasting with the findings of Wojtczak et al. (2017).

Methodological differences between the Guest et al. (2019) study and the studies of Valero et al. (2016, 2018) and Wojtczak et al. (2017) include; (a) measurement of AR threshold rather than growth, (b) ipsilateral rather than contralateral measurement of the AR, (c) use of a pure-tone elicitor rather than a BBN elicitor, and (d) the use of a conventional 226 Hz probe instead of a broadband probe to measure the response. A recent study by Mepani et al. (2019) reported that AR threshold and strength are correlated with word recognition scores in challenging listening environments. These same speech thresholds are also correlated with the summating potential/action-potential ratio, and the inference is that underlying cochlear synaptopathy drives both relations. However, this study does not factor in an estimate of noise exposure and so it is not possible to conclude whether noise exposure, and noise-induced cochlear synaptopathy, are able to account for these findings. Shehorn et al. (2020) demonstrated that a lower AR magnitude was associated with poorer speech recognition and also with higher levels of lifetime noise exposure. These data were collected on a group of listeners who had sought help for listening difficulties and those that had not.

The use of a broadband probe by some studies (Mepani et al., 2019; Shehorn et al., 2020; Valero et al., 2016, 2018; Wojtczak et al., 2017) is a potentially critical difference which requires further consideration. Although there are differences in the specific metric reported (i.e., whether it is expressed as absorbance or reflectance), these alternative approaches require that instead of a 226 Hz tone being used to measure the acoustic admittance of the ear, a click is used. This allows the energy admittance of the full frequency spectrum to be measured. Bharadwaj et al. (2019) highlight that this is a desirable approach, as if a single-frequency measure of the AR is made, it is possible to misrepresent the true underlying AR response. If the spectral characteristics of the response are different across listeners a single point-like estimate of the AR at 226 Hz could result in an inaccurate characterization of the AR. However, as can be seen by the spectra presented in Wojtczak et al. (2017), the dominant spectral frequency is 1 kHz, and these lower frequency changes in sound pressure (caused by changes in admittance and reflectance) dominate any changes seen in the higher frequency regions. Therefore, although a wideband probe does yield AR information from the full spectrum, it is still the case that this estimate is dominated by a limited number of high amplitude frequency components.

The wideband probe technique was developed primarily to broaden the applicability of the clinical technique and its diagnostic capabilities (Feeney & Keefe, 1999). The broadband probe is able to evoke thresholds at lower sound levels and so is able to yield a threshold in a larger proportion of tested patients as there is greater dynamic range before the maximum activator level is reached. This means the reflex can be used as part of the diagnostic toolbox in patients with sensorineural hearing loss or hyperacusis, and it also avoids the need to present potentially harmfully loud sounds to people (Schairer et al., 2013). There is evidence that the broadband probe stimulus leads to lower thresholds, which is clearly shown in Figure 12 of Feeney et al. (2017). This same figure indicates that the thresholds obtained via the broadband and clinical tonal probe on each individual are in broad agreement, as the relation between thresholds obtained via the different probe tones appears to be robust (though the strength of this relation is not quantified). Based on these data, the differences in AR in audiometrically normal listeners reported by Wojtczak et al. (2017) and proposed by Bharadwaj et al. (2019) should also be seen using the clinical approach. To date, there is no strong evidence that, provided a response can be measured using the clinical system, the relative strength across different listeners would be qualitatively different if a broadband probe was used. However, it remains possible that the choice of probe may account for the differences between studies, and further data are needed to resolve this issue.

The primary aim of the current study was to measure both the AR threshold and growth function in audiometrically normal listeners with varying degrees of lifetime noise exposure, using a clinical middle-ear analyzer. Most of these listeners would not have tinnitus, and so we would be able to evaluate if the AR can stratify the degree of synaptic loss in people with normal hearing and no tinnitus. Although Guest et al. (2019) measured thresholds, and only in response to ipsilateral tonal elicitors, the current study used both broadband and tonal elicitors and both contralateral and ipsilateral presentations. The hypothesis was that if the AR is a good biomarker for subclinical changes in cochlear synapses, the threshold should be elevated, and suprathreshold growth reduced, in listeners who report higher levels of lifetime noise exposure.

## Materials and Methods

The project was preregistered on the Open Science Framework before data collection began (osf.io/8ahgk). The preregistration stated that a curve would be fitted to the observed data points to allow accurate estimation of the AR threshold and growth. The type of curve was not specified, which, in retrospect, was an oversight. For simplicity, a linear function was instead used to model the data.

### Participants

Forty-eight young adults (aged 18–40 years, 30 female) were recruited into the study. This number was sufficient to detect an effect size of 1.3 (half that reported by Wojtczak et al., 2017) using an alpha of .05 with 90% power. The age range was selected to ensure participants had clinically normal audiometric thresholds. Participants were also required to show no abnormal findings on otoscopic examination of their outer ears, normal pure-tone hearing thresholds (≤20 dB HL at 0.25, 0.5, 1, 2, 3, 4, 6, and 8 kHz), and no reported history of ear surgery, neurological disorder, or head trauma. Forty-five of the 48 participants met these criteria. Three participants were excluded from the study, two due to hearing loss, and one due to atelectatic tympanic membranes. Only one person reported having tinnitus.

Of the 45 participants whose data were used in the final cohort, 43 had normal tympanometric results (middle-ear compliance 0.3–1.5 cm^3^, middle-ear pressure –50 to +50 daPa, and ear canal volume between 0.6 and 1.5 cm^3^). Two participants had hypercompliant eardrums (compliance >1.5 cm^3^); however, they were still included in the analyses as their tympanograms displayed a “Type A” shape, and there was no indication of middle-ear dysfunction. The final cohort had a mean age of 27 years (*SD* = 6 years) with 27 females. All participants gave informed written consent, and all testing materials and procedures were approved by a University of Manchester Divisional Research Ethics Committee (#4768).

### Audiometric Thresholds

Pure-tone air-conduction audiometric thresholds were measured from the right ear of all participants according to British Society of Audiology (2011) recommended procedures at conventional frequencies using a Kamplex KC50 audiometer and TDH39 headphones. Only the right ear was tested to keep the testing session short. It was assumed that given the cohort demographic, normal hearing in the right ear would typically indicate normal hearing in the left ear and that the better ear would be random across the cohort. Extended high-frequency audiometric thresholds (at 12 and 16 kHz) were also obtained using Sennheiser HDA300.

### Distortion Product Otoacoustic Emissions

Distortion product otoacoustic emissions were recorded from the right ear of all the participants using an Otodynamics Echoport ILO288. Forty-one of 45 participants (91%) had present distortion product otoacoustic emissions, defined as having a response amplitude >0 dB SPL at 3 or more test frequencies. Distortion product otoacoustic emissions were obtained to screen for gross outer hair cell dysfunction, not currently detectable using pure-tone audiometry, which could account for reduced ARs.

### AR Thresholds and Growth Functions

ARs were measured from the right ear using a GSI Tympstar diagnostic middle-ear analyzer with Grason KR-Series clinical ear tips, as this was the same ear from which audiometric thresholds were evaluated. Calibration was performed before each test session using a 2-cc coupler. Tympanograms and ARs were measured using a 226 Hz probe tone (trains of ∼40 ms pulses). Three 1.5 s elicitors—0.5 kHz pure tone, 2 kHz pure tone, and 0.4–4 kHz BBN—were presented ipsilaterally and contralaterally, creating a total of six stimulus conditions. The six conditions were tested in a pseudorandom order with each condition tested three times. The initial presentation level was 70 dB HL for tonal stimuli and 60 dB HL for the broadband stimulus. In each “run,” a reflex threshold response was first determined and then three further measurements were made at 5, 10, and 15 dB above the initial estimated threshold. The reflex threshold was defined as the lowest stimulus intensity resulting in a reduction in middle-ear compliance of ≥0.02 ml, with appropriate morphology and no evidence of significant measurement artefact. The process of measuring the threshold and the three suprathreshold reflexes was then repeated twice more before moving on to the next condition. If a significant measurement artefact was observed during a response period, the presentation was repeated.

A linear regression was performed on each of the three runs for a condition, and these functions were then averaged. Using this estimate of the AR, the stimulus level (in dB HL) at which tympanometric compliance would change by 0.02 ml was extrapolated, and this was taken as the AR threshold. The slope of the function was used to calculate the growth of the AR, and this is represented as the rate of change in compliance for each 5 dB in stimulus level (cm^3^/5-dB). We chose to express the change relative to a 5-dB stimulus level increase as we felt that this could be a more intuitive way to think of the growth change for clinicians.

### Lifetime Noise Exposure

The Noise Exposure Structured Interview (NESI; Guest et al., 2018) was used to evaluate subjects’ lifetime noise exposure (to intensities greater than 85 dBA). During the interview, participants were directed to (a) identify occupational and/or recreational noisy activities in which they had engaged; (b) for each activity, divide the life span into periods in which exposure habits were approximately stable; (c) estimate exposure duration for each period, based on frequency of occurrence and duration of a typical exposure; (d) estimate exposure level, based on vocal effort required to hold a conversation or, for personal listening devices, typical volume control setting; and (e) report usage and type of hearing protective equipment. The resulting data from all activities and life periods were combined to yield NESI units of lifetime noise exposure, a measure linearly related to the total lifetime energy of exposure above 85 dBA. Full details of the procedures are described in Guest et al. (2018). The interview was conducted by a separate researcher to the one measuring audiometric and AR data and was typically done at the end of the testing session. If it was performed at the start of the testing, the two researchers did not discuss the noise exposure status of the volunteer to eliminate any experimenter bias in the procedures.

## Results

### Hearing Thresholds

**Figure 1.**
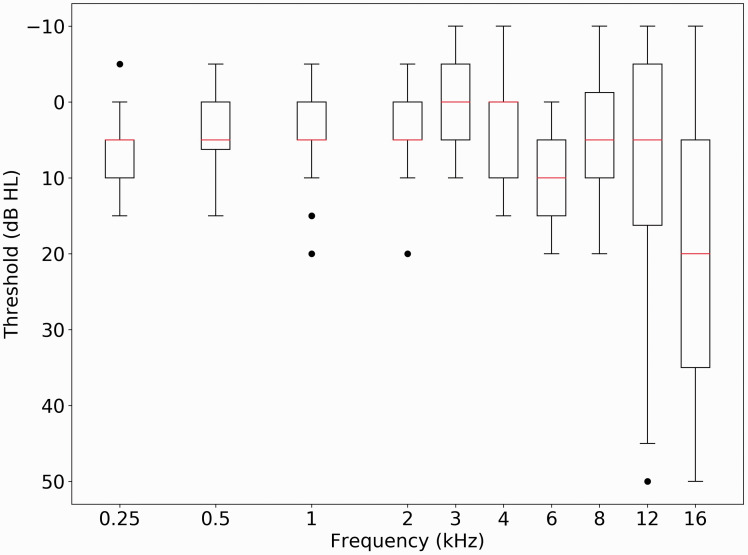
Boxplots Are Shown in dB HL for All Audiometric Frequencies Tested. The horizontal line denotes the median and the box length the interquartile range. Whiskers show the extent of the data with outliers (defined as ±1.5 × IQR) plotted as individual points.

Figure 1 shows average audiometric thresholds (and 1 standard deviation) for the 45 listeners at each audiometric frequency tested. All participants have hearing in the normal range at all frequencies up to 8 kHz.

### Noise Exposure

**Figure 2.**
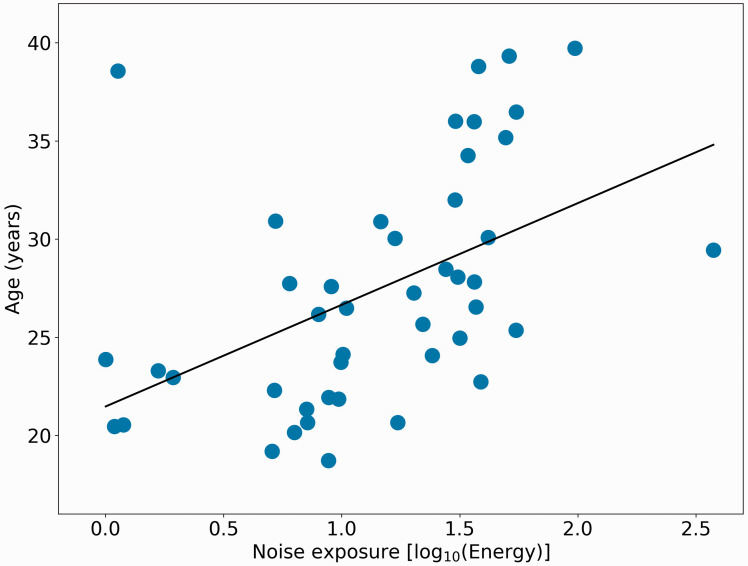
Scatterplot of Individual NESI Scores [log_10_(Energy)] for Each Participant, as a Function of the Age of Participant.

Figure 2 shows a scatterplot of noise exposure scores and age. The Pearson correlation between age and noise exposure is 0.48 (*p* < .001). Although there is a reasonable spread of noise exposure histories, there is only one person with a high exposure (>2) and only a small number with values below 0.5.

### Acoustic Reflexes

**Figure 3.**
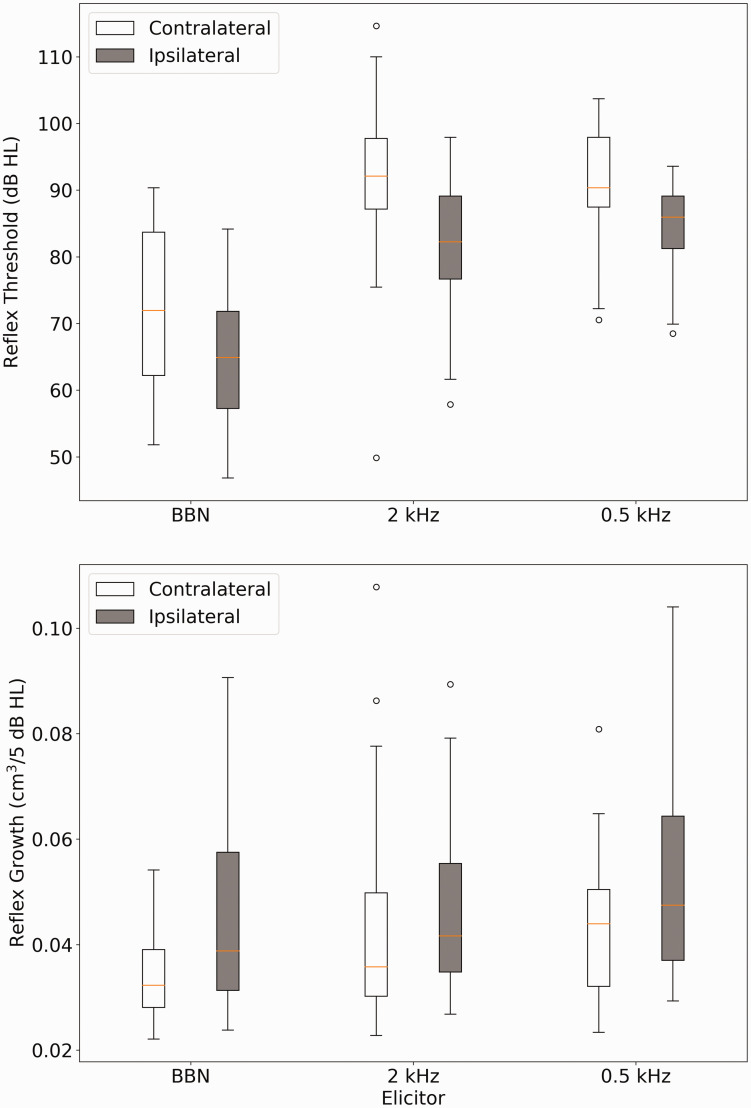
Box Plots of Acoustic Reflect Threshold (Top Chart) and Acoustic Reflex Growth (Bottom Chart) for the Six Stimulus Conditions: Broadband Noise, 2 kHz and 0.5 kHz, in Both Ipsilateral and Contralateral Stimulation. BBN = broadband noise.

Figure 3 shows the AR threshold and AR growth measurements across the group for the six conditions. This shows two expected characteristics: (a) BBN gave the lowest AR thresholds and (b) for all three elicitor stimuli, ipsilateral thresholds were 5 dB lower than contralateral ones. For some participants, it was not possible to obtain a reflex for all conditions, and Table 1 summarizes the percentage response rate and maximum elicitor output permitted by the system for all six conditions.

**Table 1.** Response Rates and the Maximum Permissible Elicitor Output Are Shown for the Six Conditions.

Ear of elicitor	Contralateral (left)			Ipsilateral (right)		
Frequency of elicitor (maximum output in dB HL)	0.5 kHz (110)	2 kHz (120)	BBN (105)	0.5 kHz (100)	2kHz (105)	BBN (105)
Response rate % (*N*)	80 (36)	98 (44)	100 (45)	73 (33)	91 (41)	91 (41)

*Note*. BBN = broadband noise.

### Planned Analyses

A two-way repeated-measures analysis of variance was performed on measures of growth, in which the factor *ear* had two levels (contralateral or ipsilateral) and the factor *elicitor* had three levels (0.5 kHz, 2 kHz, and BBN). Tests of sphericity revealed no violation of assumptions and a main effect of both ear, *F*(1, 27) = 37.41, *p* < .001, and elicitor, *F*(2, 54)=4.454, *p* = .016, with no significant interaction, *F*(2, 54) = 2.99, *p* = .059. Simple main effects analysis confirmed that there was a significant difference between the ear of elicitor presentation for BBN and 0.5 kHz elicitors.

Ipsilateral estimates of AR growth were larger than contralateral growth (*p* < .001 and *p* = .003 for BBN and 0.5 kHz elicitors, respectively). There was no difference in AR growth measured ipsilaterally or contralaterally for the 2 kHz elicitor. For contralateral presentations, AR growth was significantly greater for 2 kHz and 0.5 kHz elicitors relative to a BBN (*p* = .007 and .013, respectively). There was no difference in contralateral reflex growth for the two tonal elicitors (*p* = .28). For ipsilateral presentations, there was no difference between reflex growth for any of the elicitors (*p* > .94 for all pairwise comparisons).

**Figure 4.**
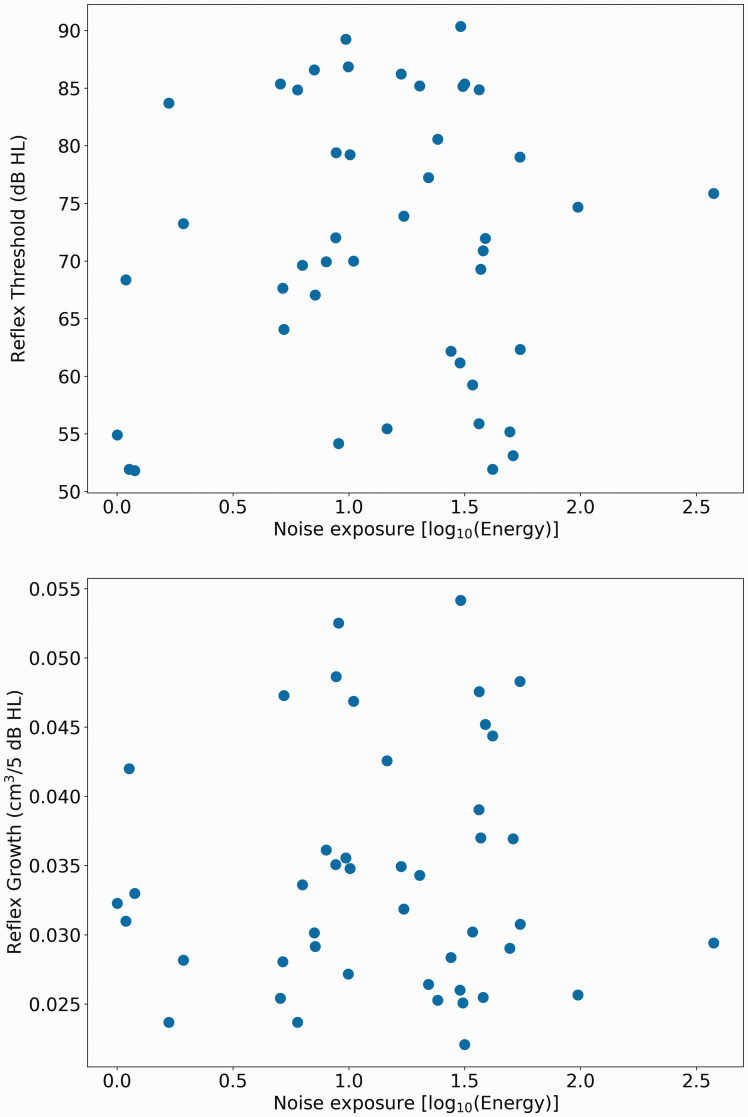
Scatterplots of Acoustic Reflect Threshold (Top Chart) and Acoustic Reflex Growth (Bottom Chart) as a Function of Noise Exposure [log_10_(Energy)].

As two separated groups of low and high noise exposure could not be identified (see preregistration for the criterion), the cohort was treated as a single group, with noise exposure as a continuous variable. The main research question concerned whether AR threshold or growth varied significantly as a function of estimated lifetime noise exposure. Figure 4 shows the relation between noise exposure and both threshold and growth for the contralateral BBN elicitor. The Spearman’s correlation coefficient for AR threshold was *r*_s_ = .009 *p* = .95 and for AR growth *r*_s_ = .074, *p* = .62.

### Exploratory Analyses

#### Reflexes and Pure-Tone Average

Although this cohort had absolute hearing thresholds ≤20 dB HL at each audiometric frequency, it is still possible that audiometric sensitivity is related to the strength of the response. To investigate this, correlation coefficients were calculated between reflex thresholds and the most relevant audiometric test frequency which were 0.5 kHz and 2 kHz for the 0.5 Hz and 2-kHz tonal elicitors, respectively. For the BBN conditions, the reflex thresholds were correlated with 1 kHz audiometric thresholds (as this is the frequency of the noise most effectively transmitted by the transducers used), the pure-tone average calculated over the range 0.25–8 kHz, and also thresholds at 16 kHz. To keep the number of comparisons down, only thresholds were considered, and contralateral conditions were used as they showed better overall response rates than the ipsilateral conditions. The correlations are shown in Table 2.

**Table 2.** Spearman’s Tests Between AR Thresholds and Specific Audiometric Thresholds Are Reported.

Contralateral elicitor	PTA frequency	Spearman’s *R*	*p* value
0.5 kHz	0.5 kHz	0.23	.19
2 kHz	2 kHz	0.17	.28
BBN	1 kHz	0.24	.11
BBN	0.25–8 kHz average	0.035	.82
BBN	16 kHz	–0.098	.52

*Note*. PTA = pure-tone average; BBN = broadband noise.

#### Agreement Between Measures

AR thresholds and growth, elicited by a contralateral BBN, showed no significant relation to each other (*r* = –.24, *p* = .12). Furthermore, as Table 3 shows, ipsilateral and contralateral measures of the threshold vary in the strength with which they are related to each other across the different elicitor stimuli. These findings suggest that the *a priori* methodological decision of whether threshold or growth will be used to quantify the reflex and whether reflexes are measured ipsilaterally or contralaterally are critical in determining how an individual’s reflex will be quantified relative to the rest of the cohort.

**Table 3.** Correlations Between the Different Measurement Conditions Are Shown for Growth and Threshold, Separately.

	Contralateral			Ipsilateral		
	BBN	2 kHz	0.5 kHz	BBN	2 kHz	0.5 kHz
Contralateral						
BBN		0.47**	0.48**	0.37*	0.37*	0.27
2 kHz	0.51**		0.44*	0.13	0.57**	0.23
0.5 kHz	0.51**	0.75**		0.18	0.61**	0.47**
Ipsilateral						
BBN	0.37*	0.61**	0.74**		0.32*	0.037
2 kHz	0.47**	0.71**	0.70**	0.55**		0.57**
0.5 kHz	0.26	0.55**	0.62**	0.55**	0.36*	

*Note*. Cells above the diagonal, shaded gray, report correlations for AR thresholds, and cells below the diagonal, with no shading, report AR growth. BBN = broadband noise.

*p ≤ .05. **p ≤ .01.

#### Middle-Ear Compliance and Reflex Strength

**Figure 5.**
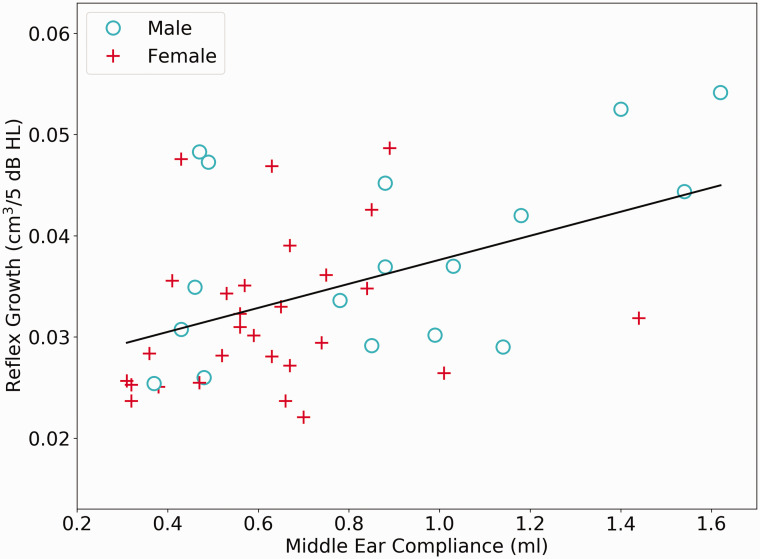
Estimates of Reflex Growth, From a Contralateral BBN Elicitor Are Plotted as a Function of Middle-Ear Compliance Values. Males are plotted as open circles and females as a plus sign.

Middle-ear compliance (the peak of the recorded tympanogram) was significantly correlated with both AR threshold (*r*_s_ = –.32, *p* = .033) and AR growth (*r*_s_ = .45, *p* = .0015) for a contralateral BBN elicitor. Therefore, the more compliant a listener’s eardrum, the lower their threshold and the steeper their growth (see Figure 5 for a plot of this relation). Although a small number of participants had hypermobile eardrums, these were not outliers on the skewed distribution of compliance values, and a nonparametric correlation coefficient was computed. Additional analysis showed that if the four listeners with compliance values above 1.2 ml are removed, the correlation with growth weakens (though remains significant) and that with threshold strengthens. If the two males with growth exceeding 0.05 cm^3^/5 db HL are removed, again the correlation with growth weakens (*p * =  .022) and that with threshold strengthens (*p* = .007).

Considering the relation between compliance and the reflex for only one elicitor is potentially misleading, and therefore, Table 4 reports the nonparametric correlations between peak tympanometric compliance and reflex threshold and growth for the six conditions. These values indicate an inconsistent relation between compliance and AR threshold, which suggests at best a weak effect. However, the steepness of the AR growth function is reliably predicted by middle-ear compliance.

**Table 4.** Spearman’s Correlation Coefficients Are Shown which quantify the relation between compliance and Reflex Growth and Threshold Across All Conditions.

	Threshold		Growth	
	Contralateral	Ipsilateral	Contralateral	Ipsilateral
BBN	–0.34*	–0.12	0.43***	0.40**
2 kHz	–0.28	–0.20	0.47**	0.47***
0.5 kHz	–0.37*	–0.26	0.57***	0.378*

*Note*. BBN = broadband noise.

*p ≤ .05. **p ≤ .01. ***p ≤ .005.

#### Sex

There was no a priori reason to expect differential AR responses between male and female listeners. However, in the cochlear synaptopathy literature, there have been questions raised over whether males and females are similarly vulnerable, given differences in their high-frequency hearing (Prendergast et al., 2017) and the fact that they do not always both show the same relation between ABR wave I amplitude and noise exposure (Stamper & Johnson, 2015a, 2015b). For contralateral AR thresholds elicited with a BBN, there was no significant difference between male (*N* = 18) and female listeners (*N* = 27; Welch’s *t* = –1.92, *p* = .063). However, for AR growth, males showed steeper functions than females (Welch’s *t* = 2.3, *p* = .028). Given the relation between compliance and AR suggested by the previous analysis, a test was also run to determine whether there was a difference in middle-ear compliance between the sexes: Welch’s *t* = 2.56, *p* = .017, indicating males show significantly higher levels of compliance.

#### Age

The existence of noise-induced cochlear synaptopathy remains a contentious and unresolved issue. However, there is growing evidence that cochlear synapses are lost across the cochlea length as the system ages (Viana et al., 2015; Wu et al., 2018). Therefore, despite the cohort being in an age range where overt age-related changes to hearing are not likely, there is value in quantifying the relation between the reflex strength and age. Nonparametric Spearman’s correlation coefficients indicate a significant positive correlation between age and lifetime noise exposure (*r*_s_ = .60, *p* < .001). There were no significant relations between age and the contralateral BBN reflex threshold (*r*_s_ = –.29, *p* = .06), reflex growth (*r*_s_ = .11, *p* = .47) or between age and peak tympanometric compliance (*r*_s_ = .07, *p* = .66).

## Discussion

### Noise Exposure and the AR

There was no evidence from the current study to suggest that, in people with normal-hearing thresholds in the conventional range, AR threshold or AR growth varied with lifetime noise exposure when using a contralateral BBN elicitor. Therefore, there is no indication that, at least with routine clinical equipment and testing paradigms, the AR is a suitable proxy for subclinical changes to the auditory system caused by noise exposure. The rationale for the study, and the underlying premise of this conclusion, assumes that an estimate of lifetime noise exposure is a reasonable proxy for the underlying degree of cochlear synaptopathy. The current study corroborates work by Guest et al. (2019), who also reported no relation between noise exposure and AR thresholds, or between tinnitus and AR thresholds. Both the current study and Guest et al. (2019) used standard clinical equipment and a 226 Hz probe tone. This was also used as one of the measures in Mepani et al. (2019), in which the results obtained with a 226 Hz probe showed the same relation with the speech measures as a custom wideband assay (although the clinical procedure did yield higher threshold values).

The current study adds to our understanding of the AR as a quantitative research tool by demonstrating that reflex growth is related to the compliance of the middle ear, and this is not equivalent between males and females. In addition, only ∼14% of the variance in ipsilateral thresholds and growth functions is accounted for by contralateral thresholds and growth; therefore, the decision over whether to observe the AR ipsilaterally or contralaterally could have a critical effect on the outcome of any study. Finally, for the most reliable elicitor (contralateral BBN, due to the fact it was the only condition to elicit a 100% response rate), estimates of threshold and growth were not reliably correlated with each other and so the choice of how to quantify the response will also lead to different estimates of underlying synaptopathy in a listener, if the AR is being used for this purpose.

Whilst this study had a range of participants with varying degrees of noise exposure, our numbers of highly exposed listeners, such as people who very frequently attend loud music events (NESI >2), were limited. Wojtczak et al. (2017) focussed on listeners with tinnitus, and so it may be that the degree of synaptopathy necessary to result in altered ARs was greater than that sustained by most listeners with normal hearing and with no symptoms of tinnitus. In the BBN condition, all our participants produced an AR, which increased with increasing elicitor level. This is in itself is different from Wojtczak et al. (2017), where a number of study participants showed no AR or little evidence of growth. It remains possible that a group of highly exposed people who do not yet have any audiometric loss may show ARs which are in alignment with those reported by Wojtczak et al. (2017). However, it may be unrealistic to expect to be able to test people who fall into this specific part of the noise-exposure continuum as it would rely on the statistical likelihood of people volunteering for the research study during the window of time that their noise exposure was sufficient to cause a measurable degree of synaptopathy but not sufficient to lead to an audiometric loss.

### Technical Considerations

As noted in the Introduction section, there are different approaches to measuring the reflex which use different probe stimuli. Whilst the conclusions drawn from the current study may be specific to the 226 Hz probe tone used, it is important for future studies to consider these issues and the extent to which they may reduce the accuracy of any measurements. Aside from the specific probe used, there are other technical considerations which may be important. For example, wideband systems typically use in-situ forward-pressure calibration to allow greater accuracy over what sound levels actually reach the tympanic membrane. Some clinical systems also offer the ability to correct presentation levels based on estimates of individual ear canal volume and physiology; however, the system we used did not. This lack of subject-specific calibration leads to increased between-subject variability which in turn will affect the power of the study. We conclude here that the clinical protocol, using a 226 Hz probe tone, is not suitable for quantitative measures of the reflex in healthy listeners, under the assumption that a subset of the cohort has sustained subclinical audiological changes. However, it remains possible that if some of these technical aspects were amended, the 226 Hz probe tone could provide sufficient statistical power across a group of listeners to quantify differences in the response related to underlying physiological changes. It may be that different tones (678 Hz, 1000 Hz, or a click) may be a better choice of probe or that different eliciting stimuli may produce less variability which is not from the source of interest (in this case, the number of cochlear synapses). However, it remains our view at the moment that any measures should look at the consistency of the response across different recording montages, assess if the reflex needs to be normalized relative to tympanometric peak compliance and if there are differences as a function of sex.

### Are Clinical and Wideband Measures Fundamentally Different?

The underlying assumption behind the work described was that if the AR is a reliable marker of cochlear synaptopathy, then, even if the wideband measure is more sensitive to detecting this, given sufficient statistical power, the clinical approach would be able to detect this effect. There are numerous examples in the literature which highlight the idiosyncratic spectral shifts which make up the reflex when using a wideband probe; for example, some listeners can have minimal reflex magnitude at 226 Hz but a very large reflex elsewhere in the spectrum. Examples such as the shifts reported in Wojtczak et al. (2017), Bharadwaj et al. (2019), and Feeney et al. (2004) all support the notion that using a wideband probe will produce a very different rank order of reflex magnitude across a group compared with the 226 Hz probe. However, there are also some instances in which a significant positive relation exists between the wideband probe measure and the 226 Hz probe across a group of listeners (Feeney et al., 2017; Schairer et al., 2007). Our assumption was that whilst the wideband probe will result in lower thresholds and a better hit-rate in listeners with no reflex using standard protocols, there is no fundamental difference between the two approaches. It may be that the wideband probe captures useful aspects of the response not available when using a single low-frequency probe. Such an account would explain the discordant findings between the present study and Shehorn et al. (2020), which were aligned in their main research questions but different in the methodological implementation. An additional difference was that our cohort consisted of normal-hearing listeners with no complaint of listening difficulty, whereas the experimental group in Shehorn et al. (2020) has sought help for perceived listening difficulties.

There are currently little available data directly comparing the two AR measures. If it were subsequently established that the two probe choices are not equivalent, then the assumptions underpinning the current study would be violated and further caution would be required when interpreting the data.

### Considerations for Future Use of the AR

The work by Wojtczak et al. (2017) and Shehorn et al. (2020) in humans, and also Valero et al. (2016, 2018) in the mouse model, has identified the AR as a potentially useful research tool in identifying subclinical changes to the auditory system in the absence of any change in absolute audiometric sensitivity. The exploratory analyses performed here highlight a number of potential issues and sources of confound which should be factored into any future study which seeks to use measures of the AR for quantitative analysis.

The measures of AR threshold and growth were found not to be correlated with each other, and therefore, it may well be of importance which measure—probe stimulus, and/or side of stimulation, is used as a dependent variable in future studies. Also, in this group of healthy listeners, a BBN elicitor produced the largest response (lowest threshold), and contralateral BBN was the only elicitor to yield a 100% response rate. However, the correlation between the ipsilateral and contralateral responses was weakest for the BBN (irrespective of whether threshold or growth is considered). Therefore, not only is it important to consider whether a measure of growth or threshold is used to quantify the response but also whether the response is measured using an ipsilateral or contralateral elicitor. The data presented in this study suggest that listeners with the strongest response in one ear might not have the strongest response using a different stimulus ear or frequency, which is a concern as this methodological choice will therefore impact the pattern of results seen.

Furthermore, middle-ear compliance was found to be a significant predictor of subsequent reflex strength for all elicitors and both measurement montages. In the current study, males showed larger levels of middle-ear compliance, and this was related to lower thresholds and steeper growth functions. Hall (1979) reported compliance values in 336 patients, and for both sexes, these values were maximal between the ages of 31–40 years. Females younger than the age of 30 showed greater levels of compliance than males younger than 30, but men showed substantially greater changes in compliance older than the age of 30, compared with females. In one of the largest studies of AR function, Jerger et al. (1972) showed higher levels of compliance for males at all ages compared with females in more than 1,000 ears. There was also a clear decrease in compliance as a function of age, though there was no difference in AR thresholds between the sexes. However, Osterhammel and Osterhammel (1979) reported data from 286 listeners and showed no sex difference for compliance or reflex strength as a function of sex. Wilson (1981) reported that measures of middle-ear compliance were not related to measures of reflex growth in different age groups of listeners. Rawool (1996) found no difference in compliance values between males and females and no relation between compliance and AR thresholds measured with a 226 Hz probe for an ipsilateral elicitor. However, there was a relation between AR thresholds measured with a 678 Hz probe for males, but not for females, though this was in the opposite direction to that reported in the current study. Fria et al. (1975) also found no relation between compliance and ipsilateral reflex threshold in a group of normal-hearing listeners. There is clearly a lack of consensus over whether the sex of a listener plays a decisive role in the strength of AR. There is also conflict in the literature over whether male and female listeners show different levels of compliance. Part of the reason for this lack of consensus may be the diverse demographics for a number of these studies, with sex ratios varying across a wide age range, which may wash out any subtle effects. Furthermore, very few studies explicitly characterize the strength of relation between compliance and AR strength and those which do typically only consider measures of threshold. Also, as Jerger et al. (1972) noted, for the early studies in this area, it was difficult to compare compliance values across different models of electroacoustic bridge. For our study, in which the listeners were all healthy and constrained within a 20-year age range, it is unclear what could have confounded the results to produce the interactions we see between compliance, growth, and sex. Therefore, we advocate that future studies consider the role of these parameters on one another.

The relation between sex, middle-ear compliance, AR threshold, and growth is unclear. Fria et al (1975) suggest that the AR (ipsilaterally elicited in their case) should be used as a qualitative measure (present or absent) as more information is needed before it can be used as a quantitative measure. Despite this statement being in excess of 40 years old, it is our conclusion that there are still too many unknown factors which influence the response for it to be used as a quantitative measure to identify subclinical hearing pathologies.

## Conclusions

There is no evidence that the AR is related to the degree of estimated lifetime noise exposure when using a 226 Hz probe. There are a number of clear experimental designs and methodological considerations which should be factored into any future studies which seek to use the AR as a quantitative research tool which are as follows:

1. The standard, clinical methodology for obtaining the AR is not appropriate for quantitative measures of the response.
2. The reflex measured may be related to the compliance of the middle ear.
3. Growth and threshold estimates of the reflex are not related to each other.
4. Ipsilateral and contralateral estimates of the response evoked with the same elicitor are not strongly predictive of each other.
